# Multifocal pleomorphic dermal sarcoma and the role of inflammation and immunosuppression in a lung transplant patient: a case report

**DOI:** 10.1186/s13256-019-2093-9

**Published:** 2019-05-30

**Authors:** Mary E. Anderson, Nemanja Rodic, Antonio Subtil, Dawn Queen, Selim Arcasoy, George W. Niedt, Peter W. Heald, Larisa J. Geskin

**Affiliations:** 1 University of Texas Southwestern Medical Center, Dallas, TX USA; 2 Department of Dermatology, Yale-New Haven Hospital, New Haven, CT USA; 3 Columbia University Vagelos College of Physicians and Surgeons, New York, NY USA; 4 Department of Dermatology, Columbia University Irving Medical Center, Herbert Irving Pavilion, 161 Fort Washington Ave, 12th floor, New York, NY 10032 USA

**Keywords:** Pleomorphic dermal sarcoma, Alpha-1-antitrypsin deficiency, Transplantation, Immunosuppression, Inflammation

## Abstract

**Background:**

Pleomorphic dermal sarcoma is the cutaneous variant of undifferentiated pleomorphic sarcoma. It is a rare malignancy of unclear histogenesis; it is a diagnosis of exclusion that requires extensive use of immunohistochemistry to rule out other malignancies. Pleomorphic dermal sarcoma typically presents as a solitary tumor in sun-exposed areas and may have unpredictable clinical behavior, with some tumors associated with metastasis and death.

**Case presentation:**

We present an unusual case of multifocal pleomorphic dermal sarcoma arising in the areas of alpha-1-antitrypsin deficiency panniculitis in a lung transplant patient. Our patient was a 58-year-old white woman whose initial presentation was consistent with alpha-1-antitrypsin deficiency panniculitis. She then developed extensive multifocal, bleeding, and ulcerated nodules in the areas of the panniculitis. A skin biopsy was consistent with a diagnosis of pleomorphic dermal sarcoma. Her immunosuppressive regimen was decreased, and she was treated with liposomal doxorubicin 40 mg/m^2^ every 3 weeks with some initial improvement in the size of her tumors. However, soon after beginning therapy, she developed pneumonia and septic shock and ultimately died from multi-organ failure.

**Conclusions:**

We hypothesize that chronic, multifocal inflammation in the skin in the setting of immunosuppression led to simultaneous, malignant transformation in numerous skin lesions. We discuss the challenges of diagnosing pleomorphic dermal sarcoma, therapeutic options, and stress the need for multidisciplinary management of these cases.

## Background

Pleomorphic dermal sarcoma (PDS) is the cutaneous variant of undifferentiated pleomorphic sarcoma (UPS). It is a rare malignancy of unclear histogenesis; it is a diagnosis of exclusion, requiring an extensive immunohistochemistry (IHC) workup to rule out epidermal, muscle, vascular, adnexal, neural, adipocyte, and melanocytic tumors [1]. PDS typically arises as a single lesion, which may progress and metastasize [2]. Chronic inflammation in the setting of immunosuppression is thought to play a role in the etiology of cancer, including sarcomas [3].

We present an unusual case of multifocal PDS arising in the areas of alpha-1-antitrypsin deficiency (A1AD) panniculitis in a lung transplant patient. We hypothesize that chronic, multifocal inflammation in the skin in the setting of immunosuppression led to simultaneous malignant transformation in numerous skin lesions. We discuss the challenges of diagnosing PDS, the therapeutic options, and stress the need for multidisciplinary management of these cases. To the best of our knowledge, this is the first report of a multifocal presentation of PDS. The unusual circumstances of this case shed light on the disease pathogenesis.

## Case presentation

A 58-year-old white woman with a history of emphysema and chronic obstructive pulmonary disease (COPD) secondary to A1AD, who received lung transplantation 4 years prior, presented to dermatology with a 1-year history of painful nodules on the extensor surfaces of her upper extremities and back. She reported a 14-year one pack/day smoking history as well as fatigue, shortness of breath, cough, allergies, arthritis, leg swelling, muscle weakness, colitis, decreased appetite, nausea, light sensitivity, eye pain, and eye redness. She also reported depression and anxiety. She denied alcohol or drug use. She had completed high school and was now supported on disability. She also received emotional support from her husband who accompanied her to appointments and was involved in her healthcare. She had a family history of a cousin with cancer (type not reported). Following lung transplantation, she had been maintained on an immunosuppressive regimen of mycophenolate mofetil (MMF), tacrolimus, intermittent steroids, and a human alpha-1 proteinase inhibitor. Her post-transplant course was complicated by multiple respiratory viral and fungal infections, recurrent acute cellular rejection and lymphocytic bronchiolitis, chronic allograft dysfunction, recurrent lower extremity deep venous thrombosis, and an intermittent requirement for increases in her immunosuppressive therapy.

**Fig. 1:**
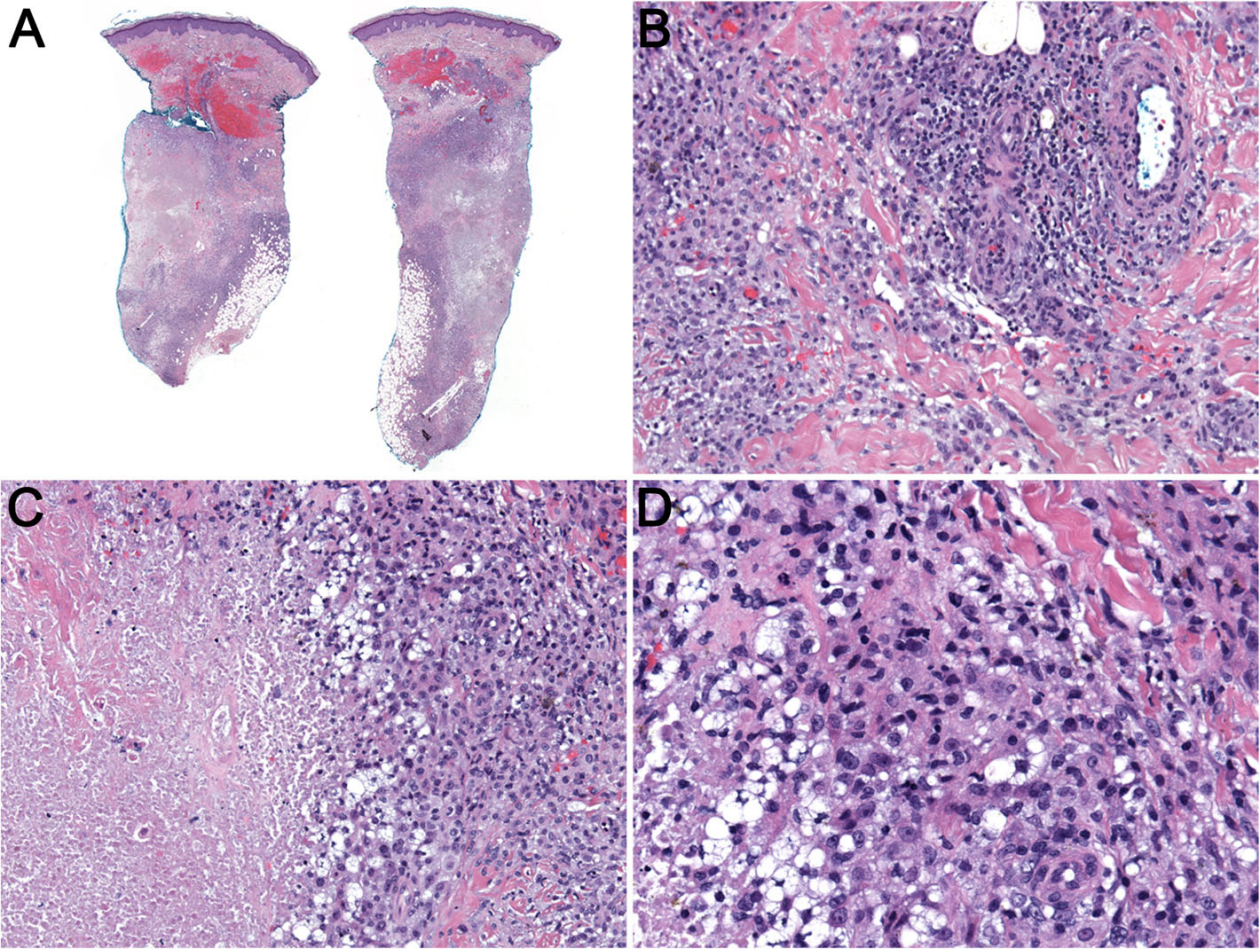
**a-d** Left lateral and right anterior lower leg biopsy. **a** Representative low-power magnification image showing granulomatous dermatitis and panniculitis involving both left lateral (*left image*) and right anterior lower leg (*right image*) biopsy. **b-d** Representative medium-power magnification images showing cellular infiltrate of lymphocytes, histiocytes, multinucleated giant cells, and areas of necrosis

Five months later, she developed multiple tender, indurated erythematous plaques on her anterior tibial surfaces bilaterally, clinically suggestive of erythema nodosum. Excisional biopsies taken from both legs showed necrotizing granulomatous dermatitis and an inflammatory infiltrate involving the panniculus (Fig. 1). An infectious workup with Gram, periodic acid–Schiff (PAS), and Fite stains did not reveal any microorganisms; there was no growth on short-term or long-term tissue cultures. A complete rheumatologic and hematologic workup, including serum protein electrophoresis (SPEP) and urine protein electrophoresis (UPEP), was unrevealing. IHC staining of the inflammatory infiltrate was positive for myeloperoxidase (neutrophilic marker), and CD68 and CD163 (histiocytic markers). A multifocal histiocytic malignancy was considered, but there were no markedly atypical cells with aberrant IHC staining to support that possibility. A repeat biopsy 1 month later showed similar findings, including presence of neutrophil-rich mixed (septal and lobular) panniculitis without vasculitis, and a diagnosis of presumptive A1AD panniculitis was made. The decision was made to increase the dose of human alpha-1 proteinase inhibitor therapy and prednisone with close follow-up.

**Fig. 2:**
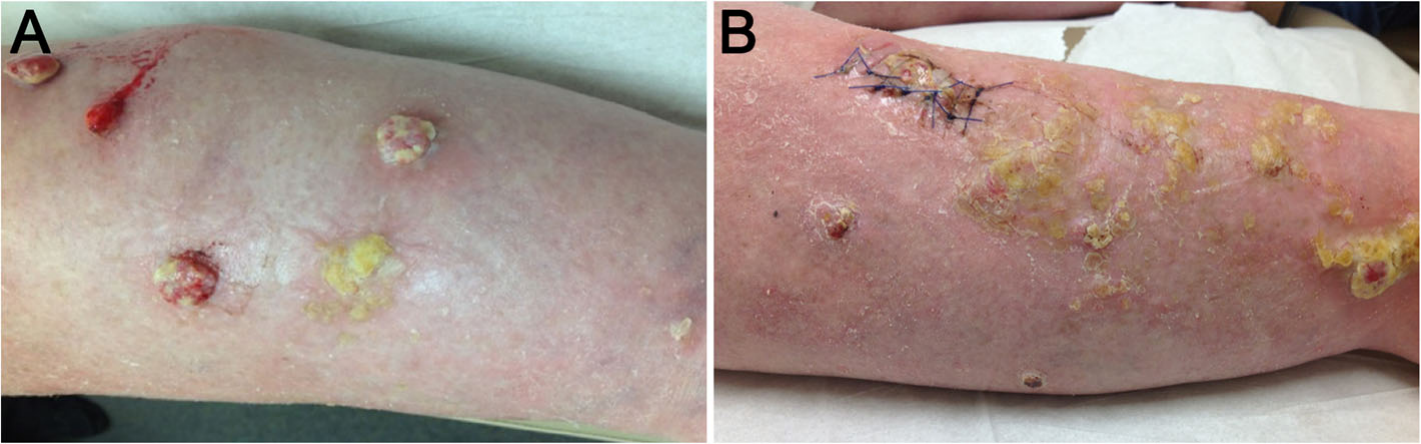
**a-b** Bilateral lower legs with multiple, exophytic bleeding, and ulcerating nodules with yellow crusting. **a** Left leg. **b** Right leg is photographed post-biopsy

**Fig. 3:**
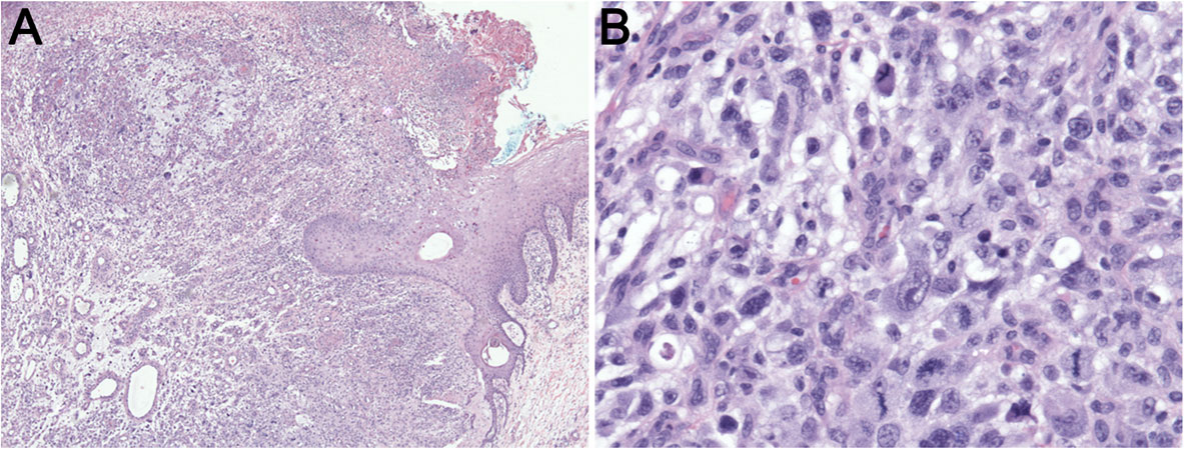
**a-b** Right anterior leg biopsy. **a** Representative medium-power magnification image showing monomorphic atypical cellular infiltrate with overlying ulceration. **b** Representative high-power magnification image showing cytologically atypical cells characterized by nucleomegaly, hyperchromasia, and frequent mitoses, including atypical forms

For the following 6 months, she was seen regularly in our dermatology clinic and noted at each visit to have significant improvement in her symptoms and lesions. However, over the course of several weeks, she developed multiple painful, exophytic, firm nodules with ulceration in the areas of panniculitis (Fig. 2). An excisional biopsy taken at that time revealed a dermal-based proliferation of large pleomorphic atypical spindle and rounded cells with marked cytological atypia, and a subset of bizarre giant cells with hyperchromatic nuclei, abundant cytoplasm, and frequent atypical mitotic figures. Focal necrosis and myxoid features were present (Fig. 3). A repeat infectious workup was negative.

IHC was negative for melanocytic (S-100, Melan-A, HMB45), epithelial (low molecular weight cytokeratin, cytokeratin MNF116, p40, p63), follicular dendritic cell (CD21), interdigitating dendritic cell (S-100), Langerhans cell (CD1a), histiocytic (CD68, lysozyme), muscle (SMA and desmin), and endothelial cell markers (CD31 and CD34). Both CD10 (positive in mesenchymal tumors, non-specific) and p16 strongly stained the pleomorphic spindle and giant cells. Together, the lack of tissue-specific markers, the histopathology findings, and strong CD10 staining indicated a diagnosis of cutaneous undifferentiated pleomorphic sarcoma (UPS)/PDS, a diagnosis of exclusion. A full body positron emission tomography-computed tomography (PET-CT) scan was notable for multifocal increased fluorodeoxyglucose (FDG) uptake in our patient’s bilateral lower extremities without the presence of distant metastatic disease.

Several therapeutic options were considered, including: cytotoxic chemotherapies commonly used in sarcoma such as doxorubicin and ifosfamide or gemcitabine and docetaxel; kinase inhibitors (such as imatinib or pazopanib); and programmed cell death-1 (PD-1) and PD-1 ligand (PD-L1) inhibitors. Although PD-1 inhibitors have generally been disappointing in sarcoma treatment, preliminary results from a phase II study suggested possible efficacy particularly in the UPS subtype [4]. In the study, of the 76 patients treated with the PD-1 inhibitor pembrolizumab, an objective response rate of 22% was noted in the UPS subgroup, while no responses were seen in any of the other sarcoma subtypes studied. However, the use of such agents in organ transplant recipients is contraindicated due to a high risk of organ rejection.

Because of the presumed relationship between immunosuppression and sarcoma, the decision was made to discontinue MMF. Considering our patient’s poor overall baseline performance status and due to concerns of myelosuppression in combination with her immunosuppressive regimen [5], she was not considered a candidate for combination therapy with doxorubicin and ifosfamide. Liposomal doxorubicin, however, has shown comparable efficacy and less toxicity compared to traditional doxorubicin and is often used for the treatment of advanced sarcoma in patients with poor performance status [6]. Given the safety profile of this combination therapy, the decision was made to proceed with liposomal doxorubicin 40 mg/m^2^ every 3 weeks.

**Fig. 4:**
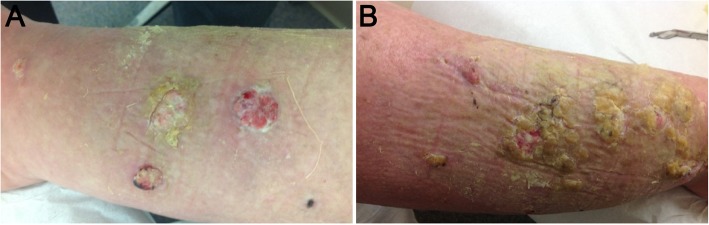
**a-b** Bilateral lower legs with reduced tumor burden following two 40 mg/m^2^ cycles of liposomal doxorubicin and discontinuation of mycophenolate mofetil. **a** Left leg. **b** Right leg

**Fig. 5:**
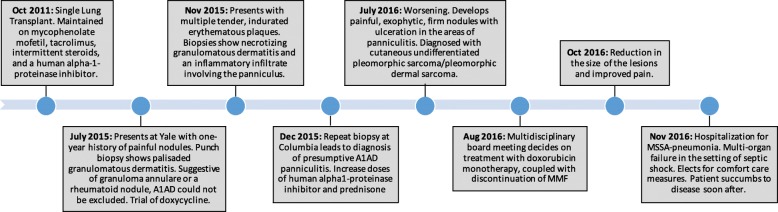
Schematic depiction of patient’s clinical course. *A1AD* alpha-1-antitrypsin deficiency, *MMF* mycophenolate mofetil, *MSSA* methicillin-susceptible *Staphylococcus aureus*

After two cycles of doxorubicin and discontinuation of MMF, our patient experienced a reduction in the size of her lesions and improved pain at her 2-month follow-up visit (Fig. 4). However, 2 weeks later, she was hospitalized with worsening fatigue, increasing dyspnea on exertion, and increasing oxygen requirements. A workup was positive for methicillin-susceptible staphylococcal aureus (MSSA) pneumonia. Acute cellular rejection or antibody-mediated rejection was also considered, although not definitively proven. Despite prompt initiation of appropriate therapy with antibiotics and steroids, her condition worsened, and she developed multi-organ failure in the setting of septic shock. She ultimately elected for comfort measures and died shortly after (timeline, Fig. 5).

## Discussion

Soft tissue sarcomas are uncommon malignancies, comprising less than 1% of all cancers [7]. UPS was first described in the 1960s as a malignant fibrous histiocytoma (MFH). The designation of MFH was assigned to a subset of soft tissue sarcomas characterized by a pleomorphic phenotype, storiform growth pattern, and unknown line of differentiation [8]. Although initially thought to be the most common form of adult sarcoma, advances in IHC and electron microscopy allowed for the reclassification of many of these “unclassifiable” tumors: one study that reviewed previously diagnosed “MFH” found that only 13% met criteria for the diagnosis [7]. Ten years later in 2002, the World Health Organization moved to eliminate the term MFH altogether, instead preferring the more phenotypically accurate term “undifferentiated pleomorphic sarcoma not otherwise specified” [9].

PDS is the cutaneous variant of UPS [10]. Given the rarity of the disease, literature on the pathogenesis, epidemiologic, clinical, and prognostic features of PDS is scarce. However, several generalizations may be made. The disease seems to occur more frequently in elderly, white patients, and slightly more often in males. The classic clinical presentation is that of a solitary, rapidly growing tumor on the head and neck [11, 12]. Bleeding and ulceration of tumors is common [11]. Although there are limited data on the prognosis of PDS, metastatic disease and death have been reported, and the morbidity and mortality of the disease may be greater than previously thought [2].

While disease pathogenesis remains unclear, immunosuppression has been proposed as an independent risk factor for aggressive PDS [13]. Both immunosuppression and chronic inflammation are well-known drivers of oncogenic cellular changes; one possible explanation is that advancing age is associated with aberrant expression of pro-inflammatory molecules and decreased immune surveillance [14]. Advanced age is also associated with low-level chronic inflammation and a decline in naive T cells critical to tumor surveillance [15]. The fact that these tumors occur more commonly in the elderly supports the role of inflammation and immunosuppression in the pathogenesis of PDS.

In addition to the epidemiologic evidence, recent investigations into PDS and A1AD revealed increased expression of chronic, pro-inflammatory molecules, including 8-nitroguanine, 8-oxo-7,8-dihydro-2′-deoxyguanosine (8-oxodG), cyclooxygenase 2 (COX-2), nuclear factor-κB (NFKB), and inducible nitric oxide synthase (iNOS) [16]. There is also a known association between A1AD and malignancy, and imbalances between alpha-1-antitrypsin and elastase such as those seen in A1AD may lead to persistent inflammation and tissue damage that promote carcinogenesis via chronic activation of the tissue necrosis factor signaling pathway [17]. The chronic inflammation seen in A1AD and other inflammatory disorders, may, in turn, lead to paradoxical immunosuppression and tumor development [18]. Our patient’s history of immunosuppression and chronic inflammation secondary to A1AD panniculitis illustrates the key role these two factors play synergistically in the development of PDS.

UPS/PDS remains a diagnosis of exclusion because of a lack of tumor-specific markers, including genetic rearrangements or signature mutations. There is no standard IHC panel used to make or exclude a diagnosis of UPS/PDS. However, one suggested algorithm emphasizes that the tumor should be negative for the presence of melanocytic, epithelial, muscle, and vascular markers [19]. On histological examination, PDS is characterized by the presence of atypical spindle and epithelioid cells in the dermis, with extension into the subcutaneous tissue and often beyond, to the fascia and muscle. These atypical cells demonstrate increased mitotic activity with ulceration, tumor necrosis, and perineural and lymphovascular invasion commonly seen on microscopy.

Some biopsies may demonstrate myxoid stromal changes, desmoplastic stromal response, pseudoangiomatous or hemorrhagic features, and osteoclast-like giant cells [11]. These features are not specific to UPS/PDS and the differential diagnosis is broad: similar findings may be found in other tumors such as leiomyosarcoma, sarcomatoid squamous cell carcinomas (sSCCs), angiosarcoma, and spindle cell melanoma. Our patient’s history of solid organ transplant and her unusual multifocal presentation also raise the diagnostic possibility of de-differentiated sSCCs or eruptive keratoacanthomas (KAs). However, the tumor stained negative for p40, an IHC epidermal marker that is both sensitive and specific and allows pathologists to distinguish sSCCs and KAs from other spindle cell tumors with a high degree of accuracy [20].

To the best of our knowledge, this is the first reported case of multifocal PDS in a lung transplant recipient, a presentation that highlights the role of immunosuppression in the pathogenesis of UPS/PDS. There is a strong precedent for both inflammation and immunosuppression leading to oncogenic cellular changes [14]. We postulate that our patient’s bilateral, persistent lower extremity A1AD panniculitis and chronic venous stasis created a pro-inflammatory environment that led to malignant transformation of her histiocytic infiltrate. Her history of solid organ transplantation and subsequently impaired cellular immune surveillance probably played a role in the development of disease and may account for its unusually aggressive and extensive nature.

Few studies have reported the prevalence of PDS in the immunosuppressed patient population. In one cohort study, 642 renal transplant recipients were monitored over a 2-year period for the development of PDS; two patients developed disease [12]. In contrast, a single-center, retrospective review of 603 renal transplant recipients found no cases of PDS [21]. A recent study utilizing the Surveillance Epidemiology and End Results (SEER) program database found an increased standardized incidence ratio for PDS in patients with chronic lymphocytic leukemia [22]. Furthermore, immunosuppressed patients may have a more aggressive course and an increased risk of mortality [13]. Interestingly, preliminary results from the phase II study of the PD-1 inhibitor pembrolizumab in soft tissue sarcomas showed signs of efficacy in UPS not observed for other sarcoma subtypes, possibly implicating a particularly important role for immunosuppression in UPS tumorigenesis.

The therapeutic challenges associated with the management of multifocal pleomorphic sarcoma demonstrate the need for multidisciplinary management of such cases. Dermatologists and pathologists play critical roles in the diagnosis of this rare cutaneous malignancy. Moreover, dermatologists play a key role in providing proper skin care and keeping inflammation at a minimum in diseases like A1AD that have the potential to drive malignant conversion through stimulating a pro-inflammatory milieu. For localized disease, coordination with surgeons is necessary: first-line therapy is surgical resection with the goal of obtaining negative margins, with or without adjuvant radiation [23]. For multifocal disease or metastatic disease, systemic therapy is the primary modality.

Traditionally, doxorubicin is the most commonly used agent for the management of soft tissue sarcomas. The benefits of combination therapy with doxorubicin and ifosfamide are less clear [24]. Recently, a phase II randomized study compared doxorubicin monotherapy with the combination of doxorubicin and olaratumab, a novel monoclonal antibody targeting the platelet-derived growth factor receptor alpha, a receptor tyrosine kinase shown to be important in sarcomagenesis [25]. Median overall survival was 26.5 months for the combination versus 14.7 months for doxorubicin, a significant improvement which led to regulatory approval for combination treatment in sarcoma. Notably, approximately 20% of patients on that study had the UPS subtype. Doxorubicin plus olaratumab will likely replace doxorubicin plus ifosfamide for the treatment of most advanced sarcomas in good performance status patients. Coordinating and balancing adjustments to the immunosuppressive regimen is also an important part of the multidisciplinary approach to these cases. Although reduction in immunosuppressive therapy may play an important role in the treatment of cutaneous UPS/PDS, it may result in an increased risk of transplant rejection [13].

## Conclusion

This is the first reported case of multifocal cutaneous UPS. The case highlights the difficulties in making the diagnosis of UPS, therapeutic challenges, and the need for a multidisciplinary approach to such cases. Most significantly, our patient’s history of chronic A1AD panniculitis and immunosuppression points to the potential synergistic role of inflammation and immunosuppression as the key drivers in the pathogenesis of this disease and its severe presentation, with inflammation-induced immunosuppression possibly predisposing stromal or dendritic cells to malignant cellular de-differentiation.

## Abbreviations

8-oxodG: 8-oxo-7,8-dihydro-2′-deoxyguanosine
A1AD: Alpha-1-antitrypsin deficiency
COPD: Chronic obstructive pulmonary disease
COX-2: Cyclooxygenase 2
FDG: Fluorodeoxyglucose
IHC: Immunohistochemistry
iNOS: Inducible nitric oxide synthase
KA: Keratoacanthoma
MFH: Malignant fibrous histiocytoma
MMF: Mycophenolate mofetil
MSSA: Methicillin-susceptible *Staphylococcus aureus*
NFKB: Nuclear factor-κB
PAS: Periodic acid–Schiff
PD-1: Programmed cell death-1
PD-L1: Programmed cell death-1 ligand
PDS: Pleomorphic dermal sarcoma
PET-CT: Positron emission tomography-computed tomography
SEER: Surveillance Epidemiology and End Results
SPEP: Serum protein electrophoresis
sSCC: Sarcomatoid squamous cell carcinoma
UPEP: Urine protein electrophoresis
UPS: Undifferentiated pleomorphic sarcoma
